# Multiomics profiling identifies the poor prognostic role of a tumor cluster with GNA15 overexpression in triple-negative breast cancer

**DOI:** 10.3389/fimmu.2025.1659183

**Published:** 2025-09-22

**Authors:** Guixin Wang, Junming Cao, Chenglu Lu, Yu Cao, Shuo Wang, Ziyi Chen, Zhaohui Chen, Yingxi Li, Yue Yu, Yao Tian, Xin Wang

**Affiliations:** ^1^ the First Department of Breast Cancer, Tianjin Medical University Cancer Institute and Hospital, National Clinical Research Center for Cancer, Tianjin, China; ^2^ Key Laboratory of Cancer Prevention and Therapy, Tianjin, China; ^3^ Tianjin’s Clinical Research Center for Cancer, Tianjin, China; ^4^ Key Laboratory of Breast Cancer Prevention and Therapy, Ministry of Education, Tianjin Medical University, Tianjin, China; ^5^ Department of Pathology, Tangshan People’s Hospital, Tangshan, Hebei, China; ^6^ Department of Urology, Affiliated Hospital of Zunyi Medical University, Zunyi, Guizhou, China; ^7^ Department of Thoracic Oncology, Tianjin Lung Cancer Center, Key Laboratory of Cancer Prevention and Therapy, Tianjin’s Clinical Research Center for Cancer, Tianjin Medical University Cancer Institute and Hospital, Tianjin Medical University, Tianjin, China; ^8^ Health Science Center, Ningbo University, Ningbo, Zhejiang, China; ^9^ Immunology Department, Key Laboratory of Immune Microenvironment and Disease (Ministry of Education), Tianjin Medical University, Tianjin, China; ^10^ Department of Thoracic Surgery, The Affiliated LiHuiLi Hospital of Ningbo University, Ningbo, Zhejiang, China; ^11^ Department of General Surgery, Tianjin Medical University General Hospital, Tianjin Key Laboratory of Precise Vascular Reconstruction and Organ Function Repair, Tianjin General Surgery Institute, Tianjin, China

**Keywords:** triple-negative breast cancer, scRNA-seq, tumor microenvironment, GNA15, heterogeneity

## Abstract

**Background:**

Tumor heterogeneity impacts invasive behaviors, treatment response, and clinical outcomes in triple-negative breast cancer (TNBC). However, this heterogeneity remains incompletely characterized. This study aims to utilize multi-scale data to investigate inter-tumoral heterogeneity and identify potential TNBC biomarkers.

**Methods:**

Single-cell RNA expression profiles were analyzed using R packages. Specifically, the infercnv, Pyscenic, GeneNMF, SCP, Vector, CellChat, and hdWGCNA packages were employed to identify malignant cells and characterize heterogeneity in transcription factors, metaprograms, lineage evolution, developmental trajectories, cell–cell interactions, and co-expression networks. Bulk RNA datasets were incorporated to assess the prognostic value of cell clusters and candidate genes. G Protein Subunit Alpha 15 (*GNA15*) expression was determined via reverse transcription–quantitative PCR (RT–qPCR) and immunohistochemistry. Cell functional assays were performed to evaluate proliferation, migration, and invasion capabilities.

**Results:**

A total of 14,335 malignant cells were isolated from epithelial cells across 15 single-cell RNA samples. Six tumor cell clusters were identified, which exhibited distinct prognoses, biological functions, driver transcription factors, and co-expression networks. Notably, the S2 cluster demonstrated association with multiple malignancy-related pathways and inferior survival outcomes. *GNA15* emerged as the S2 cluster hub gene. *In vitro* experiments confirmed that *GNA15* knockdown significantly attenuated proliferation, migration, and invasion in TNBC cell lines.

**Conclusions:**

Our study comprehensively delineated TNBC tumor cell heterogeneity and established the critical role of *GNA15* in TNBC progression. These findings enhance the understanding of TNBC heterogeneity and provide a theoretical foundation for TNBC treatment.

## Introduction

Breast cancer has become the leading cause of cancer-associated disease in women worldwide for decades (1, 2). As a heterogeneous disease, its high heterogeneity contributes to differential treatment responses, recurrence patterns, and metastatic potential (3). With advances in precision medicine, biomarkers including estrogen receptor (ER), progesterone receptor (PR), Erb-B2 receptor tyrosine kinase 2 (HER2), and Ki-67 have guided molecular subtyping and therapeutic strategies, substantially improving clinical management (4, 5). However, triple-negative breast cancer (TNBC) lacks ER, PR, and HER2 expression, restricting treatment options primarily to chemotherapy (6). As the most heterogeneous subtype of breast cancer, TNBC demonstrates more aggressive biological behavior than other subtypes, resulting in poorer prognoses and earlier metastasis (7). Hence, characterizing the intra-tumoral heterogeneity and disease progression process of TNBC is crucial for improving the prognosis of TNBC patients.

The emergence of sequencing technology has accelerated the exploration of tumor heterogeneity (8). Jiang et al. (9) classified TNBC into four subtypes—mesenchymal-like, basal-like immune-suppressed, immunomodulatory, and luminal androgen receptor subtypes—using transcriptomic and genomic data. In addition, Lehmann et al. (10) used expression profiles to identify six distinct TNBC subtypes: one mesenchymal stem-like, one immunomodulatory, two basal-like subtypes, one mesenchymal, and one luminal androgen receptor subtype. These studies revealed TNBC heterogeneity at the tissue level, informing therapeutic approaches. However, tumor progression represents a continuous process wherein transcriptional characteristics vary among tumor cells within individual patients. Single-cell RNA sequencing (scRNA-seq) technology represents a powerful tool for resolving cellular heterogeneity in TNBC (11, 12). Numerous single-cell studies have characterized immune cell heterogeneity. For example, Savas et al. (13) employed scRNA-seq to explore the role of CD8 tissue-resistant T cells and confirmed their favorable role and clinical benefit for early-stage TNBC. Zhang et al. (14) elucidated the important role of mast cells in TNBC immune checkpoint therapy via single-cell sequencing. However, the single-cell research of TNBC tumor cells mainly focuses on its cell subclonal heterogeneity and evolution (15). Translational research targeting tumor cell heterogeneity, including the identification of biomarkers and the development of clinical application tools, remains to be further explored. The combined analysis of different omics offers multi-scale perspectives for cancer research and enhances the value of clinical transformation.

In this study, we successfully isolated malignant cells from epithelial populations and classified six tumor clusters exhibiting distinct transcriptional profiles, metaprograms, and molecular functions. Importantly, the S2 tumor cluster demonstrated a more aggressive phenotype, correlating with poor prognosis. The pro-tumorigenic effect of the S2 cluster appears mediated by intrinsic lineage evolution and interactions with tumor-associated fibroblasts. Furthermore, we identified G Protein Subunit Alpha 15 (*GNA15*) as the hub gene of the S2 cluster. *In vitro* experiments confirmed that *GNA15* drives proliferation, migration, and invasion in TNBC cells. Collectively, this work systematically delineates tumor cell heterogeneity across transcription factors, lineage evolution, and co-expression regulatory networks at single-cell resolution and provides the first documentation of the pro-tumorigenic role of *GNA15* in TNBC. Our findings advance the understanding of TNBC intra-tumoral heterogeneity and propose a novel therapeutic target for TNBC treatment.

## Methods

### Data acquisition and sample selection

This study incorporated four public datasets: three scRNA-seq cohorts and one bulk RNA sequencing cohort. Fifteen TNBC scRNA-seq profiles were sourced from the Gene Expression Omnibus (GEO) database (GSE176078, GSE199515, and GSE180286), and the remaining profiles diagnosed with other molecular subtypes were excluded. Bulk RNA-seq profiles and corresponding clinical information were obtained from The Cancer Genome Atlas (TCGA) using the “TCGAbiolinks” (16) package. Gene expression matrices were converted to transcripts per million (TPM) format to normalize for gene length and sequencing depth. TNBC sample inclusion criteria were as follows: 1) availability of both expression profiles and complete survival data and 2) TNBC confirmation via immunohistochemical staining. The final cohort comprised 121 TNBC samples and 113 normal breast tissue samples. Additionally, a total of 319 TNBC samples (the METABRIC cohort) with expression profiles were downloaded from the cBioPortal database (https://www.cbioportal.org/).

### Data preprocessing of scRNA-seq

A total of 15 scRNA-seq samples were downloaded (**Supplementary File**: **Supplementary Table S1**), and strict quality control standards were adopted to incorporate high-quality single cells for subsequent analysis using the “Seurat” (17) package (v4.3.0, v5.2.1). The single-cell matrix expression profile was standardized using the glmGamPoi algorithm. The specific inclusion criteria were as follows: 1) nFeatures_RNA > 200, 2) nCount_RNA > 500, 3) the proportion of mitochondrial genes < 20%, and 4) the proportion of hemoglobin genes < 1%. As potential doublets inevitably exist in single-cell sequencing, the “Doubletfinder” (18) package (v0.2.3) was utilized to remove doublets with the doublet ratio set as 0.8%. The batch effect among samples was removed using the “Harmony” (19) package, and the number of cell clusters at distinct resolutions was identified using the Louvain algorithm with the assistance of the “Clustree” package (v0.5.0) when needed. The similarity of the expression profiles of cells was visualized using a non-linear dimensionality reduction algorithm [Uniform Manifold Approximation and Projection (UMAP)]. After the single-cell standard quality control process, the distinct cell clusters were annotated based on public research and the CellMarker database (20, 21).

### Copy number variation analysis

A copy number variation (CNV) analysis was performed to identify malignant cells from total epithelial cells. Specifically, the myeloid cells were extracted as reference cells for benign cells, while all the epithelial cells were used as observed cells. The “AnnoProbe” package was used to annotate the start and end positions of genes. The CNV score was calculated using the “infercnv” (https://github.com/broadinstitute/inferCNV) package (v1.14.2). Then, the top 50% observed cells of the CNV score were selected to calculate the correlation between them and the observed and reference cells (22). Ultimately, malignant cells were defined as CNV score > 0.001 and CNV correlation > 0.5.

### Non-negative matrix factorization analysis

Non-negative matrix factorization is an unsupervised learning method widely used in the decomposition of complex data (23). The “GeneNMF” (https://github.com/carmonalab/GeneNMF) package (v0.4.0) was utilized to identify the metaprogram of tumor cells. The top 1,000 highly variable genes were identified using the “FindVariableFeatures” function. The K-value was set as 4 to 9. When the number of programs was set as 6, the metaprograms (MPs) were stable. The represented genes of distinct MPs were subjected to an enrichment analysis using the “fgsea” package. The signature scores of MPs were calculated using the “Ucell” (24) package.

### Transcription factor analysis

The gene matrix of tumor cells was transformed into loom format. A transcription factor analysis was performed using pySCENIC (v0.12.1). “motifs-v10nr_clust-nr.hgnc-m0.001-o0.0.tbl” was used as motif annotation. “hg38_10kbp_up_10kbp_down_full_tx_v10_clust. genes_vs_motifs.rankings.feather” was used as an input file to infer the correlation strength between each gene and known motif. The “SCENIC” package was used to visualize the output results.

### Pseudo-time and enrichment analysis

The “Vector” (25) package was utilized to infer the cell developmental origin. Then, the pseudo-time cell trajectory was analyzed using the slingshot method, which is an unsupervised algorithm. An enrichment analysis of each tumor cluster was performed using the “ClusterGVis” (https://github.com/junjunlab/ClusterGVis) package (v0.1.2). To be specific, the top marker genes were identified as log2FC > 0.25 and min.pct > 0.25. The biological and molecular functions of each cluster were investigated based on the gene sets collected by the Gene Ontology (GO) and Kyoto Encyclopedia of Genes and Genomes (KEGG) databases.

### *In silico* cell cytometry and survival analysis

To infer the abundance of tumor clusters in TCGA-BRCA cohort, the single-cell RNA matrix of tumor clusters was extracted, non-repetitive random sampling was adopted, and 400 cells were selected from each cell cluster as the reference matrix. The TNBC samples obtained from TCGA cohort and the METABRIC cohort were used to infer the matrix. The absolute mode was selected. CIBERSOFTX (26) was used to calculate cell abundance. The analysis of the options was set to the default parameters. After the intersection of the cell abundance score file and survival information, the “survminer” package was used to determine the optimal cut-off value of distinct cell abundance. Log-rank test was used to evaluate the statistics of the overall survival outcome. *p* < 0.05 was considered statistically significant.

### High-dimensional weight co-expression network and cell–cell communication analyses

The “hdWGCNA” (27) package (v0.2.18) was used to construct the weighted gene co-expression network analysis (WGCNA) for tumor clusters. The genes expressed in at least 5% cells were included. The metacells were constructed to reduce the sparsity of the single-cell expression matrix. The soft threshold was set as 9, and 19 modules were identified. Additionally, the cell–cell communication among cell types was investigated using the “CellChat” (28) package (v1.6.1). The receptor–ligand pairs were collected using “CellChatDB.human”. Data preprocessing involves identifying highly expressed genes and highly expressed receptor–ligand pairs.

### Bulk RNA sequencing and online database analysis

Normal breast (N=113, TCGA cohort) and TNBC tissues (N=121, TCGA cohort) were downloaded, and a differentially expressed gene analysis was performed using the “limma” (29) package (v3.54.2). The genes with *p* < 0.05 and logFC > 0.5 were considered overexpressed. Also, a survival analysis was performed based on the optimal value of each gene using the “survminer” package (v0.4.9). Gene set enrichment analysis (GSEA) of *GNA15* was performed using the “clusterProfiler” (30) package (v4.6.2). Concretely, the samples were divided into high and low groups based on the median value of gene expression. Differentially expressed gene results between the high and low *GNA15* groups were used as an input file for GSEA. The expression level of *GNA15* in distinct molecular subtypes of breast cancer was evaluated using the “UALCAN” database (31). *In silico* drug screening was performed using the Connectivity Map (https://clue.io/).

### Breast cancer specimens

The specimens of triple-negative breast cancer confirmed by pathology were obtained from Tianjin Medical University Cancer Institute and Hospital. The study was approved by the Ethics Committee of Tianjin Medical University Cancer Institute and Hospital (Ek2021021), and written informed consent was obtained from the participants.

### Cell culture and transfection

Breast cancer cell lines MCF-7, T47D, MDA-MB-231, and Cal-51 were obtained from American Type Culture Collection (ATCC; Manassas, VA, USA). MCF-7 and MDA-MB-231 were cultured in dulbecco’s Modified Eagle Medium (DMEM) (Gibco, Grand Island, NY, USA), while T47D and Cal-51 were cultured in 1640 (Gibco, Grand Island, NY,USA). All media included 10% fetal bovine serum (FBS; Vazyme, Nanjing, China) and 1% penicillin/streptomycin (Gibco, USA). The cells were maintained at 37°C in a humidified 5% CO_2_ atmosphere in a cell incubator. SiRNAs targeting *GNA15* constructs were synthesized by GentleGen (Suzhou, China), and the oligonucleotide sequences are listed in **Supplementary File**: **Supplementary Table S2**.

### Cell function assays

For the Cell Counting Kit-8 (CCK-8) assay, cells were seeded in 96-well plates at 2 × 10^3^ cells/well. On days 1–5, 100 µL of CCK-8 solution was added to each well and incubated in the dark for 1 hour. Absorbance was measured at 450 nm using a microplate reader (Bio-Rad Laboratories, Hercules, CA, USA). For colony formation assays, 5 × 10^2^ cells/well were seeded in six-well plates and cultured until colonies formed. Colonies were fixed with 4% paraformaldehyde, stained with 1% crystal violet, imaged, and quantified. For the cell scratch assay, transfected cells were cultured in six-well plates for 24 hours to form confluent monolayers. A straight scratch was created in each well using a sterile pipette tip. Cells were maintained in serum-free medium to eliminate FBS influence on migration. Scratch images were captured at 0 and 24 hours under a light microscope (Olympus, Tokyo, Japan). The migration distance was quantified as the ratio of the distance at 24 hours to that at 0 hours and was statistically analyzed. For the Transwell invasion assay, Matrigel-coated inserts (BD Biosciences, San Jose, CA, USA) were used. Briefly, 5 × 10^4^ cells in serum-free medium were seeded into the upper chambers, with medium containing 20% FBS as a chemoattractant in the lower chambers. After 14–24 hours of incubation, invaded cells were fixed and stained using a commercial kit (Thermo Fisher Scientific, Waltham, MA, USA). Cells were counted under a light microscope (Olympus, Tokyo, Japan) at ×20 magnification.

### Immunohistochemistry and antibodies

An immunohistochemistry (IHC) analysis was performed for GNA15 on TNBC tissue using a rabbit anti-*GNA15* antibody (1:250, 12078-1-AP; Proteintech, Wuhan, China) with the EnVision two-step procedure (21). *GNA15*-positive samples were defined as those with brown staining in the cytoplasm. IHC scoring was assessed by two independent, experienced pathologists. *GNA15* staining intensity was rated as follows: 0 (negative), 1 (weak yellow), 2 (moderate yellow), or 3 (strong brown). The percentage of positive cells was scored as follows: 0 (0%), 1 (1%–25%), 2 (26%–50%), 3 (51%–75%), or 4 (>75%). The final IHC score (range, 0–12) was calculated by multiplying the intensity score by the percentage score. Final scores between 1 and 3 of staining were considered low expression, while final scores between 4 and 8 were considered median expression. Final scores between 9 and 12 were considered high expression.

### Reverse transcription–quantitative PCR analysis

Total RNA was isolated from tissues and cells using the SPARKeasy Cell/Bacterial RNA Kit (Sparkjade, Shandong, China) according to the manufacturer’s instructions. RNA quality and concentration were assessed using a NanoDrop 2000 spectrophotometer (Thermo Fisher Scientific, USA). For mRNA analysis, reverse transcription–quantitative PCR (RT–qPCR) was performed using the All-in-One First-Strand cDNA Synthesis SuperMix for qPCR (TransGen Biotech, Beijing, China). GNA15 and GAPDH primers were synthesized by GentleGen (Suzhou, China), and all specific sequences are listed in **Supplementary File**: **Supplementary Table S3**.

### Statistical analysis

All bioinformatics analyses were performed using RStudio (v4.2.2 and v4.3.3), and the related statistics could be found in the corresponding section in the Methods section. For experimental assays, Student’s t-test was used for comparisons between two groups. Data were presented as mean ± standard deviation (SD), and *p* < 0.05 was considered significant.

## Results

### The characterization of tumor microenvironment landscape of TNBC

**Figure 1:**
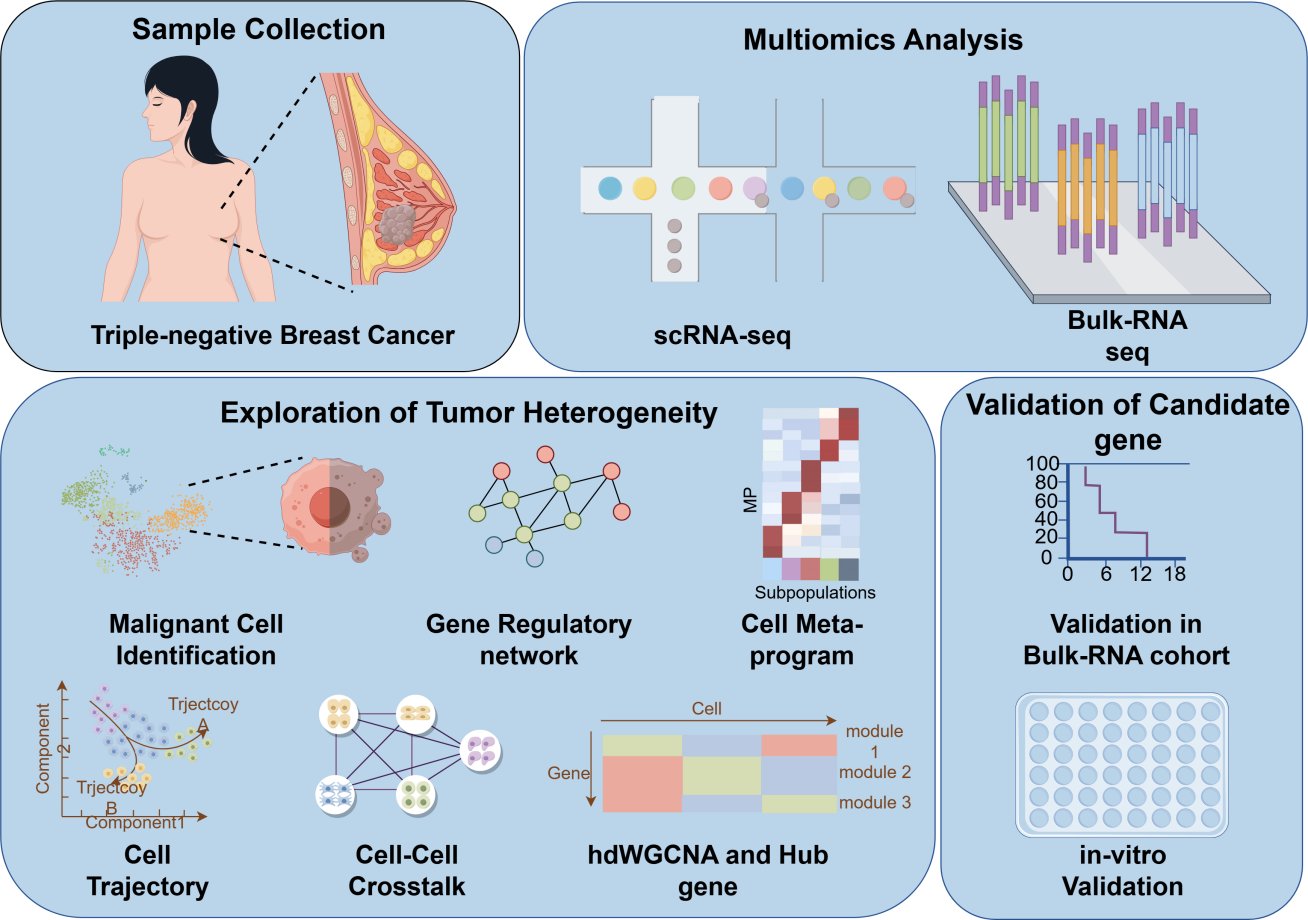
Flow chart of the study design. scRNA-seq, single-cell RNA sequencing; hdWGCNA, high-dimensional weighted gene co-expression network analysis.

**Figure 2:**
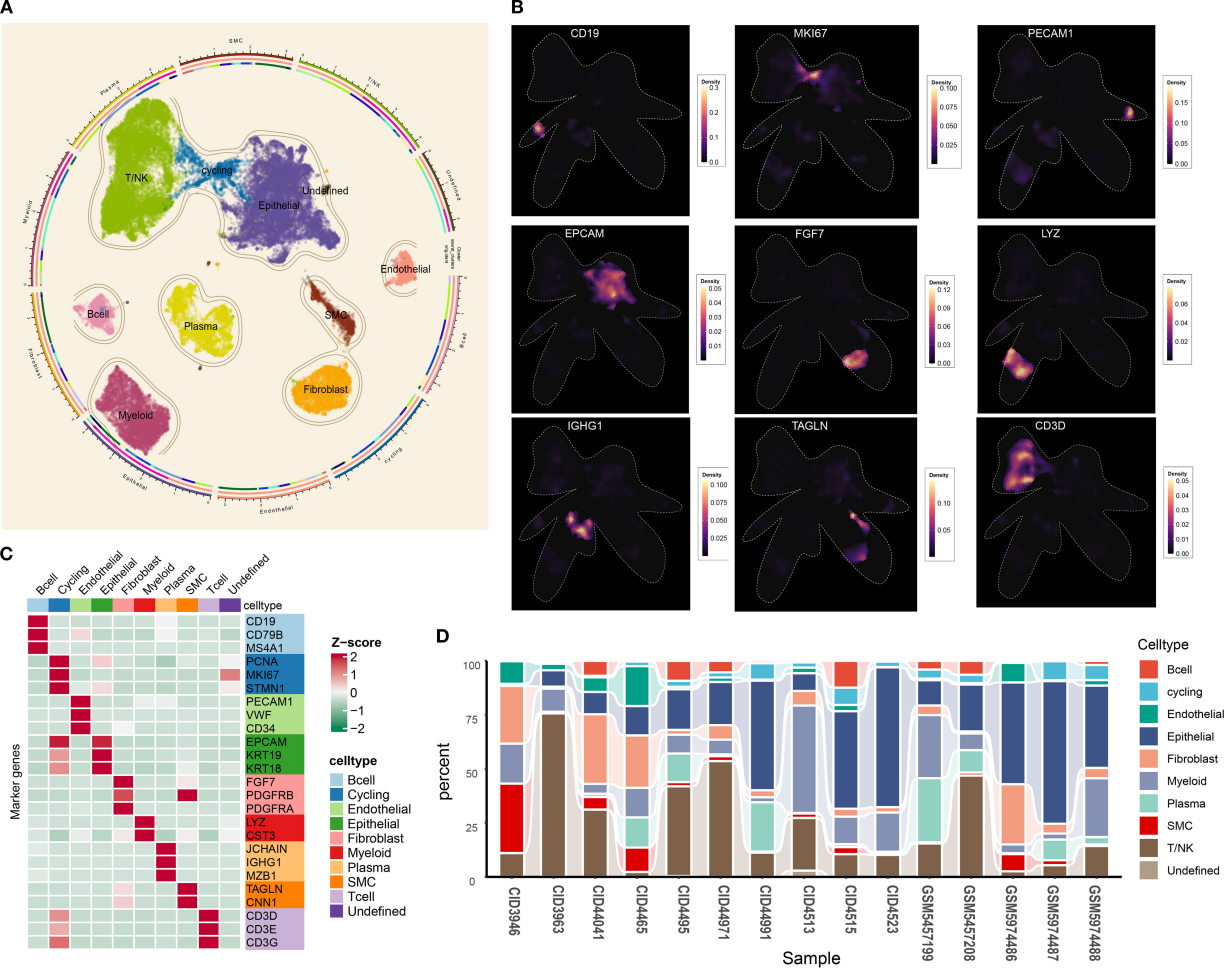
Tumor microenvironment landscape of triple-negative breast cancer. **(A)** UMAP scatter plots displaying nine different cell clusters in TNBC samples. **(B)** The density of cells with specific marker was illustrated using UMAP. The color represents the density level of the cells with the marker. **(C)** The markers used for distinct cell type identification. **(D)** The abundance of nine main cell types in TNBC samples. UMAP, Uniform Manifold Approximation and Projection; TNBC, triple-negative breast cancer.

The overall design of the study is presented in **Figure 1**. To obtain high-quality single cells, the “DoubletFinder” package was employed to remove the doublets for each sample (**Supplementary Figure S1**). Subsequently, a large single-cell matrix was constructed by integrating samples, and the batch effect among samples was removed (**Supplementary Figure S2**). After preprocessing, a total of 54,867 cells with 29,259 features were obtained for further analysis. Twenty-four cell clusters were delineated. Using established markers, nine principal cell types were annotated: cycling cells, B cells, epithelial cells, endothelial cells, fibroblasts, T/NK cells, smooth muscle cells, plasma cells, and myeloid cells (**Figure 2A**). Cells lacking definitive markers were designated as undefined. UMAP visualization confirmed robust segregation of distinct cell types (**Figure 2B**). All markers utilized for annotation are cataloged in **Figure 2C** (e.g., B cells: *CD19*, *CD79B*, and *MS4A1*; epithelial cells: *EPCAM*, *KRT18*, and *KRT19*). As shown in **Figure 2D**, cell proportions of each sample were characterized, and the results showed that the tumor microenvironment of TNBC was highly heterogeneous among different samples. Collectively, a nine-cell-type framework was established for subsequent investigation, and pronounced inter-sample heterogeneity was observed in TNBC.

### Malignant cell identification

**Figure 3:**
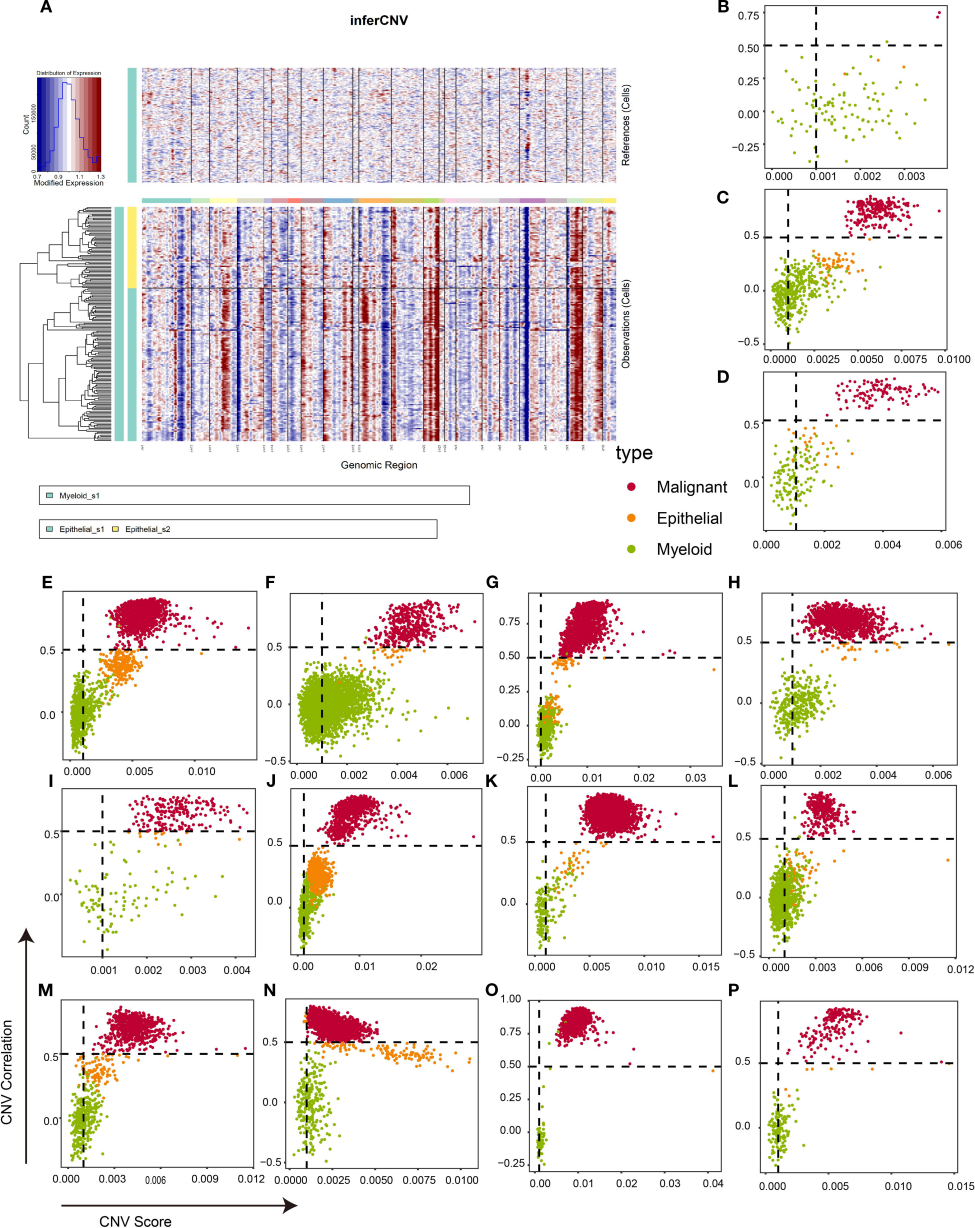
Identification of malignant cells in TNBC tissues based on infercnv algorithm. **(A)** An example of infercnv algorithm showing chromosomal landscape of inferred CNVs among epithelial cells. **(B–P)** Scatter plot showing the CNV correlation and CNV score of each sample in CID3946 **(B)**, CID3963 **(C)**, CID4465 **(D)**, CID4495 **(E)**, CID4513 **(F)**, CID4515 **(G)**, CID4523 **(H)**, CID44041 **(I)**, CID44971 **(J)**, CID44991 **(K)**, GSM5457199 **(L)**, GSM5457208 **(M)**, GSM5974486 **(N)**, GSM5974487 **(O)**, and GSM5974488 **(P)**. TNBC, triple-negative breast cancer; CNVs, copy number variations.

Epithelial cells are widely regarded as the origin of breast cancer cells (32). However, distinguishing tumor cells from epithelial populations solely through biomarker expression at single-cell resolution remains challenging. Therefore, the “inferCNV” package was used to infer the tumor cells for each sample. As illustrated in **Figure 3A**, epithelial cells exhibited significant chromosomal amplifications and deletions relative to myeloid reference cells. The CNV correlation and CNV score were calculated to identify malignant cells. As shown in **Supplementary Figure S3**, the CNV level of myeloid cells is almost concentrated in CNV score < 0.001 and CNV correlation < 0.5. Based on these results, tumor cells in epithelial cells were defined as a CNV score > 0.001 and CNV correlation > 0.5, and the remaining epithelial cells with a similar CNV level to myeloid cells were defined as normal epithelial cells. **Figures 3B–P** confirm that this threshold robustly segregated myeloid cells from malignant populations across all samples, with normal epithelial cells exhibiting intermediate CNV characteristics. In summary, 14,335 tumor cells were isolated for downstream analysis.

### Characterization of the heterogeneity of tumor cells in TNBC microenvironment

**Figure 4:**
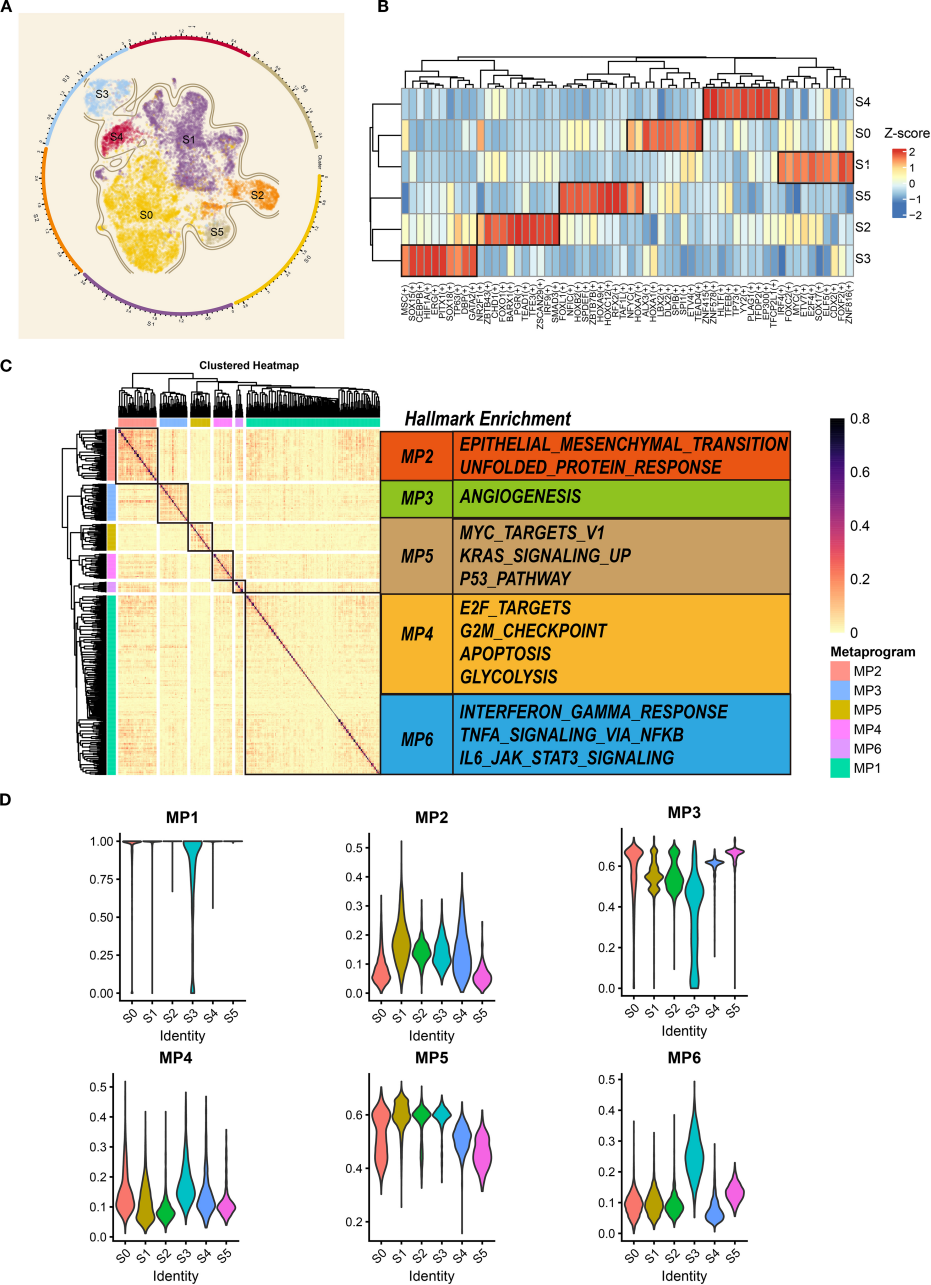
The gene patterns of each tumor cluster. **(A)** UMAP scatter plots displaying six different tumor clusters in TNBC samples. **(B)** The heatmap showing the top 10 transcription factors in each cluster. **(C)** The non-negative matrix showing five different metaprograms of tumor clusters at mRNA level. **(D)** Violin plots exhibiting the signature score of each metaprogram in distinct tumor clusters. UMAP, Uniform Manifold Approximation and Projection; TNBC, triple-negative breast cancer.

Tumor heterogeneity impacts the biological behavior, treatment response, and prognosis of tumors (33). Consequently, resolving tumor cell heterogeneity is critical for advancing TNBC precision medicine. Following high-confidence malignant cell identification, we integrated, re-normalized, and dimensionality-reduced tumor cells using established methods. Six tumor clusters were resolved: S0 (N=5,858), S1 (N=5,070), S2 (N=1,248), S3 (N=1,013), S4 (N=709), and S5 (N=436) (**Figure 4A**). A transcription factor (TF) analysis identified the top 10 most active TFs per subgroup (**Figure 4B**), revealing significant inter-cluster heterogeneity (**Figure 4B**). For example, *TEAD4* and *ETV* exhibited high activity in the S1 cluster, which indicated that the cluster may be associated with stem-like breast cancer. Also, *FOXO1*, *TEAD1*, and *SMAD3* showed high activity in the S2 cluster, suggesting that the S2 cluster may be correlated with more aggressive biological behaviors. These findings demonstrate intrinsic transcriptional heterogeneity across TNBC subpopulations.

Next, we constructed the stable MPs to further explore the heterogeneity of tumor clusters. As shown in **Figure 4C**, six metaprograms (representing coordinated biological programs) were identified. Concretely, MP2 was associated with the tumor metastasis (epithelial–mesenchymal transition), while MP5 and MP4 were correlated with cell proliferation (E2F targets, G2M checkpoint, MYC targets, and p53 pathway). MP3 was associated with tumor microenvironment formation (angiogenesis), and MP6 correlated with immune response (interferon-gamma response, TNFA signaling, and *IL6*–*JAK*–*STAT3* signaling). Interestingly, the genes of MP1 have not been enriched in any pathway. Of note, we observed that the MP2 signature score was higher in the S1 and S2 clusters, indicating that these clusters may promote tumor metastasis (**Figure 4D**). Also, the tumor proliferation-related metaprogram MP5 signature score was higher in the S1, S2, and S3 clusters. However, the other tumor proliferation-related metaprogram MP4 signature score was observed to be higher in the S3 and S4 clusters. This suggests heterogeneous proliferative mechanisms within the TNBC microenvironment. All in all, these analyses elucidate functional divergence among tumor subpopulations and their mechanistic drivers, providing a framework for targeted therapeutic development.

### Characterization of tumor cell trajectory

**Figure 5:**
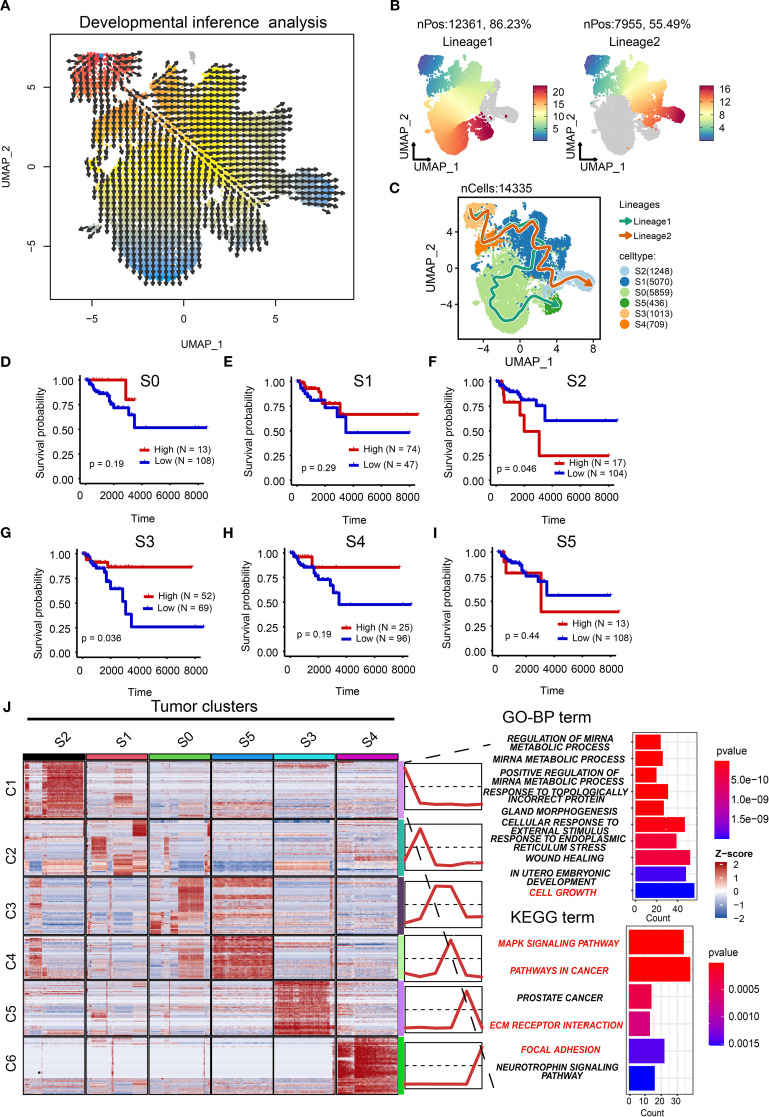
Characterization of cell trajectory. **(A)** Vector representation of developmental directions for tumor cells. **(B)** Two cell lineages of tumor cells with pseudo-time development. **(C)** The potential cell trajectory and lineages of tumor cells in tumor microenvironment. **(D–I)** The Kaplan-Meier (K-M) curves showing the overall survival rate of high and low S0 **(D)**, S1 **(E)**, S2 **(F)**, S3 **(G)**, S4 **(H)**, and S5 **(I)** tumor clusters. **(J)** Heatmap showing the mean of top 50 marker genes of clusters. The line graph represents the differential expression of the mean of these marker genes in all clusters. The bar chart represents the functional enrichment of GO (BP, biological process; CC, cellular component; MF, molecular function) and KEGG pathways for marker genes in tumor cluster S2. GO, Gene Ontology; KEGG, Kyoto Encyclopedia of Genes and Genomes.

The plasticity of tumor cells is an important characteristic for their adaptation to microenvironmental changes (34). We therefore sought to reconstruct developmental lineages across tumor clusters. First, pseudotemporal ordering inferred cellular origins (**Figure 5A**), establishing the S3 cluster as the putative root state. A cell trajectory analysis showed two potential cell lineages (**Figure 5B**). Concretely, the S3 cluster may be the initial state of tumor cells, while the S4 and S1 clusters may be the intermediate states of tumor cells. The difference is that the S0 and S5 clusters are the final states of lineage 1, while the S2 cluster is the final state of lineage 2 (**Figure 5C**).

Prognostic validation using TCGA-BRCA cohort demonstrated that the root S3 cluster exhibited favorable survival (*p*=0.036; **Figures 5D–I**), whereas the terminal S2 cluster showed significantly poorer outcomes (*p*=0.046). This confirms the clinical relevance of our trajectory model and positions S2 as a high-priority therapeutic target. The poor prognostic role of the S2 cluster was also validated in the METABRIC cohort (**Supplementary Figure S4**). Therefore, it is necessary to explore the potential biological function of the S2 cluster. We analyzed the top marker genes of each cluster as well as associated pathways. As illustrated in **Figure 5J**, GO and KEGG analyses showed that the S2 cluster was associated with cell growth (**Supplementary File**: **Supplementary Tables S4**, **S5**), the MAPK signaling pathway, pathways in cancers, extracellular matrix (ECM) receptor interaction, and focal adhesion, indicating that the S2 cluster may drive tumor progression through proliferative, metastatic, and microenvironment-remodeling mechanisms. Taken together, we establish S2 as a pivotal driver of TNBC aggressiveness.

### Cell–cell communication between tumor clusters and other components in tumor microenvironment of TNBC

**Figure 6:**
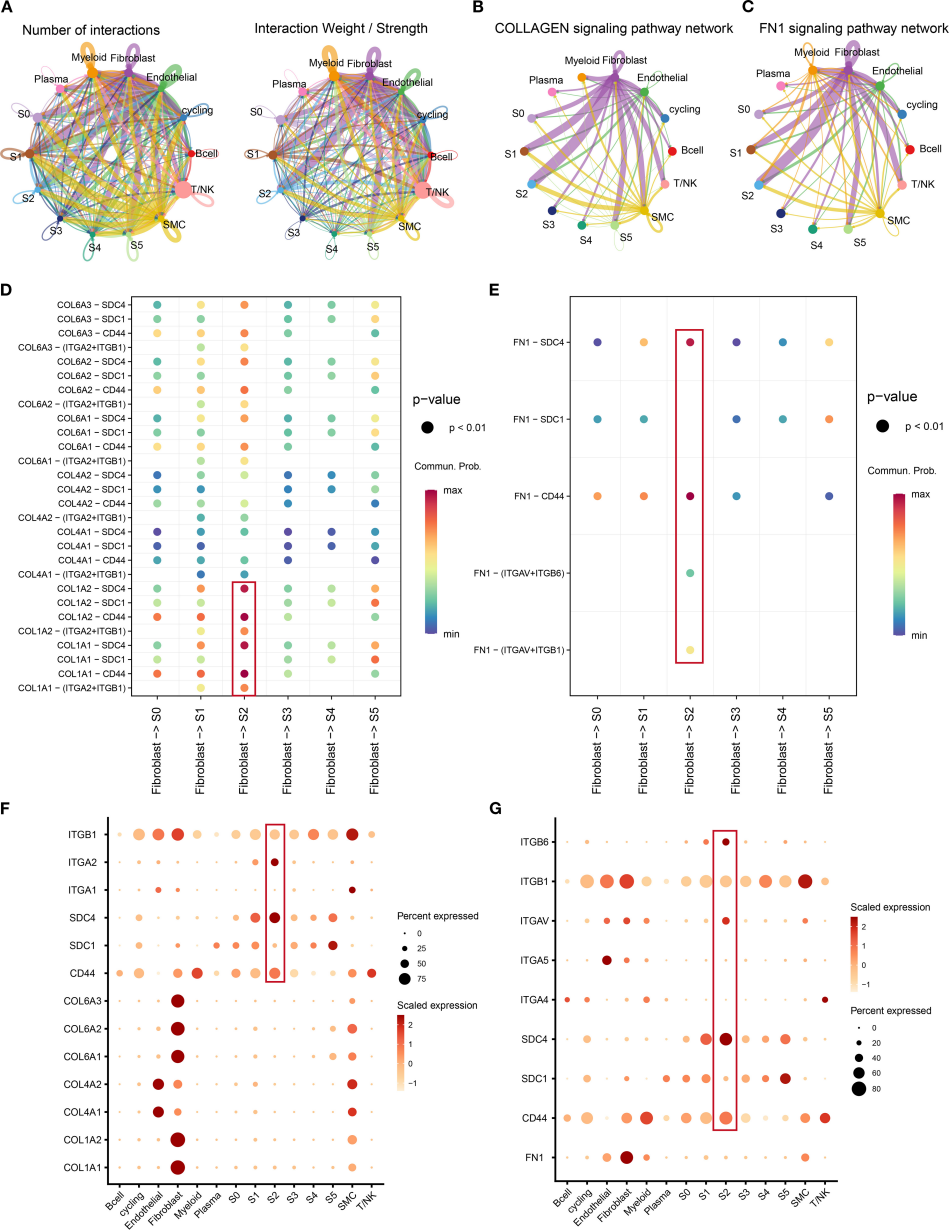
The crosstalk between tumor clusters and other components in tumor microenvironment. **(A)** The number of interactions and interaction weight among all cell types in TNBC microenvironment. The thickness of the lines represents the strength of the communication. **(B, C)** The pathway network of collagen **(B)** and FN1 **(C)** in tumor microenvironment. The thickness of the lines represents the strength of the communication. **(D, E)** Circle plot demonstrating the communication of collagen **(D)** and FN1 **(E)** signaling pathways between tumor clusters and cancer-associated fibroblasts. A color change from blue to red indicates an increase in communication probability. Dots from small to large represent smaller *p*-values. **(F, G)** The dot plots showing the expression level of collagen **(F)**, FN1 **(G)**, and associated genes among all cell types. A color change from light red to deep red indicates an increase in gene expression. Dots from small to large represent an increase in the percentage of gene expression. TNBC, triple-negative breast cancer.

The interactions between tumor clusters and other components have been confirmed to play a critical role in tumor progression (35). Beyond intrinsic evolution, we hypothesized that S2 cluster behavior may be modulated by extrinsic factors. The “CellChat” package was used to estimate the interactions and strength in the tumor microenvironment of TNBC (**Figure 6A**). We noticed a significant interaction between fibroblasts and the S2 cluster. Combining the previous KEGG results (**Figure 5J**), we focus on the interaction of ECM-related ligand–receptor pairs between fibroblasts and the S2 cluster. Fibroblasts and the S2 cluster exhibited pronounced enrichment in the collagen and FN1 pathways (**Figures 6B, C**). Key collagen ligand–receptor pairs (*COL1A2-CD44*, *COL1A2-SDC4*, *COL1A1-SDC4*, and *COL1A1-CD44*) demonstrated high communication probability (**Figure 6D**). Similarly, *FN1* pairs (*FN1-CD44* and *FN1-SDC4*) showed elevated interaction likelihood (**Figure 6E**). Fibroblasts, as signal initiators, highly expressed ligands *COL1A1*, *COL1A2*, and *FN1* (**Figures 6F, G**). Conversely, S2 (signal receiver) highly expressed receptors *CD44*, *SDC4*, *ITGAV*, *ITGA2*, and *ITGB6*. Of note, *CD44*, *ITGAV*, *ITGA2*, and *SDC4* were confirmed to be upstream of the PI3K/AKT or MAPK pathway (35–37). The results revealed the potential molecular mechanism between tumor clusters and cancer-associated fibroblasts. These results elucidate a molecular mechanism whereby cancer-associated fibroblasts (CAFs) regulate the S2 cluster through ECM receptor signaling, synergistically promoting malignant phenotypes beyond intrinsic evolutionary trajectories.

### Hub gene identification for S2 cluster

**Figure 7:**
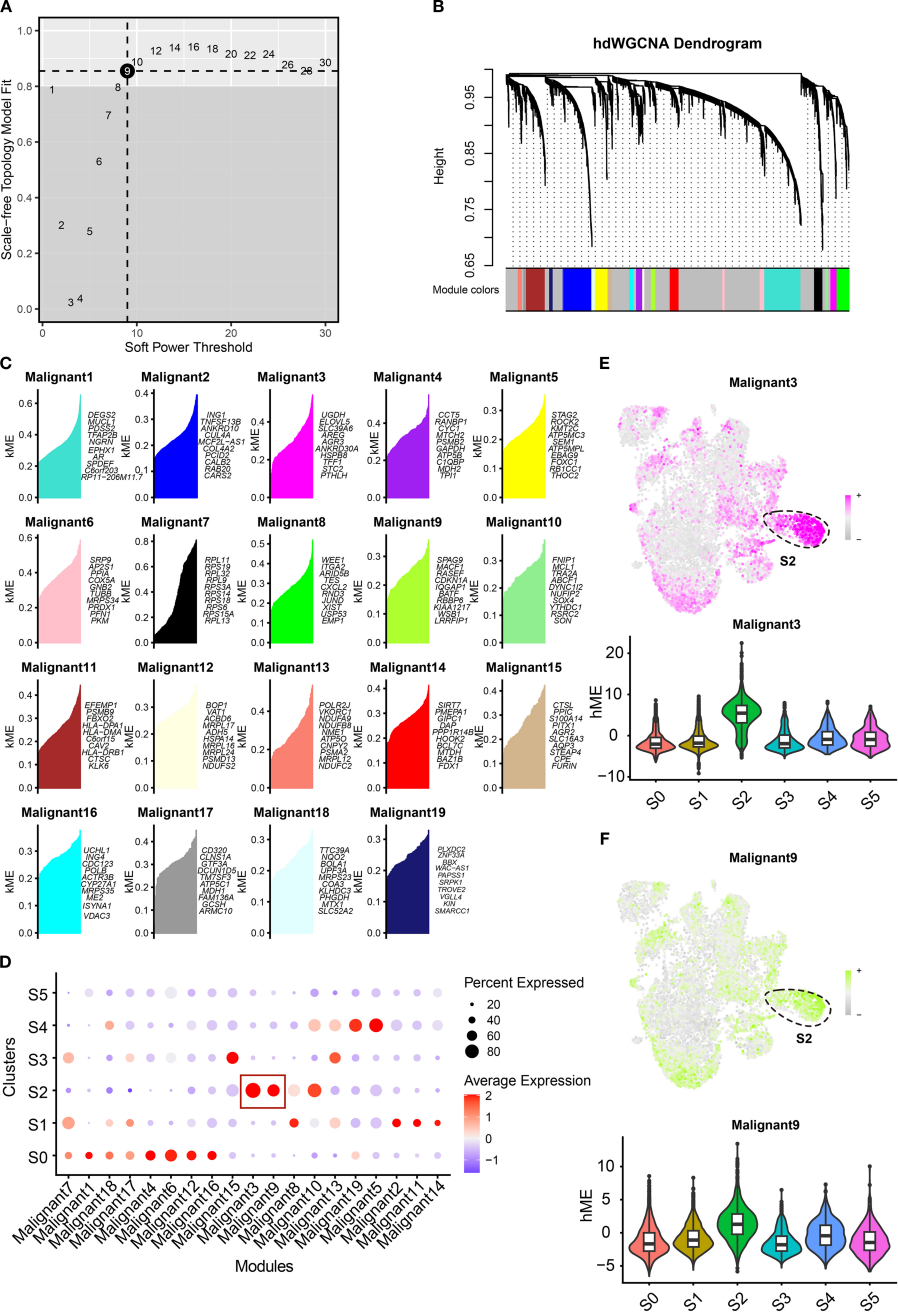
Construction of gene co-expression modules for TNBC clusters. **(A)** The optimal soft threshold was determined by weighted gene co-expression network analysis. **(B)** The hdWGCNA dendrogram showing 19 modules obtained. **(C)** Dot plots showing the signature score of distinct modules in each cluster. **(D, E)** The UMAP scatter and violin plots showing the score of malignant 3 **(E)** and malignant 9 **(F)** in tumor clusters. TNBC, triple-negative breast cancer; hdWGCNA, high-dimensional weighted gene co-expression network analysis; UMAP, Uniform Manifold Approximation and Projection.

Given the pivotal role of the S2 cluster in the TNBC tumor microenvironment, identifying therapeutic targets is imperative for improving patient outcomes. We constructed a high-dimensional weighted gene co-expression network analysis (hdWGCNA) to identify cluster-specific co-expressed genes using single-cell RNA expression profiles. The network achieved optimal connectivity at a soft threshold power of 9 (**Figure 7A**). Nineteen co-expression modules were resolved, with the top 10 hub genes of each module cataloged in **Figures 7B, C**. Module signature scores, calculated using the Ucell package, revealed marked enrichment of modules 3 and 9 specifically in the S2 cluster (**Figure 7D**), suggesting that their genes represent hub candidates for this aggressive subpopulation. UMAP visualizations and violin plots corroborated these findings (**Figures 7E, F**). Collectively, we identified 271 hub genes for the S2 cluster. By integrating these with prior transcription factor analyses, we constructed a comprehensive molecular regulatory network for this subpopulation. These results establish a framework for elucidating TNBC tumor cell heterogeneity and advancing targeted therapies.

### GNA15 identified as a key gene for S2 cluster

**Figure 8:**
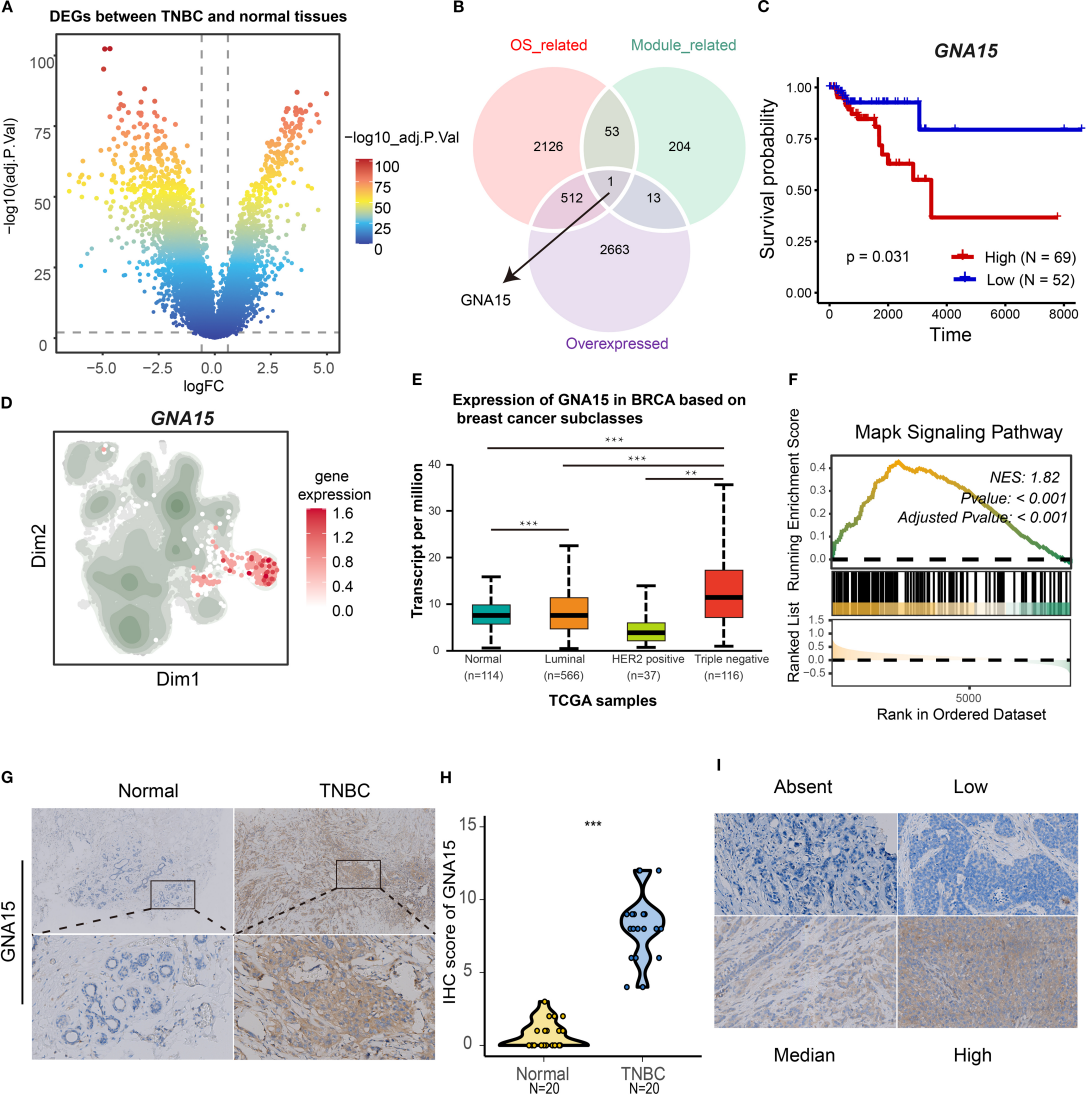
Identification of GNA15 as a core gene for S2 cluster. **(A)** Volcano map showing the differentially expressed genes between normal and TNBC patients based on TGCA-BRCA cohort. **(B)** The Venn plot showing the intersection of overexpressed genes, overall survival-associated genes, and module genes (malignant 3 and 9). **(C)** The K-M curves showing the overall survival rate of high and low *GNA15* groups in TNBC. **(D)** The expression level of *GNA15* in tumor clusters. **(E)** The boxplot showing the mRNA expression level of *GNA15* in distinct breast cancer types and normal breast tissues. **(F)** GSEA plot showing the enrichment of MAPK signaling pathways associated with high expression level of *GNA15*. **(G, H)** The protein expression level of *GNA15* in 20 paired TNBC tissues was detected by IHC, and the scores of IHC staining were obtained. ***p < 0.001. **(I)** The standard score of IHC staining. TNBC, triple-negative breast cancer; GSEA, gene set enrichment analysis; IHC, immunohistochemistry.

To identify therapeutic targets and biomarkers for TNBC, we refined candidate genes through sequential filtering to establish core drivers and validate analytical accuracy. Differential gene analysis revealed TNBC-overexpressed genes versus normal breast tissue (**Figure 8A**). Subsequent survival analysis identified genes correlating with poor prognosis. The integration of overexpressed genes, module-associated genes (**Supplementary File**: **Supplementary Table S6**), and survival-linked genes pinpointed *GNA15* as the S2 cluster core gene (**Figure 8B**). Elevated *GNA15* expression correlated with significantly poorer overall survival (log-rank *p* = 0.031; **Figure 8C**). *GNA15* was also overexpressed in the S2 cluster and TNBC tissues (**Figures 8D, E**). Gene set enrichment analysis confirmed that high expression of *GNA15* was associated with the MAPK signaling pathway (**Figure 8F**, Normalized Enrichment Score (NES) = 1.82, adjusted *p*-value < 0.001), which was consistent with the biological function of the S2 cluster. Protein-level validation via immunohistochemistry demonstrated marked *GNA15* upregulation in TNBC versus normal tissues (**Figures 8G, H**). The standard stain score of IHC is illustrated in **Figure 8I**. These results suggest *GNA15* as a core regulatory element of the S2 cluster and a promising biomarker for TNBC progression.

### Downregulation of GNA15 suppresses proliferation, migration, and invasion of TNBC cells

**Figure 9:**
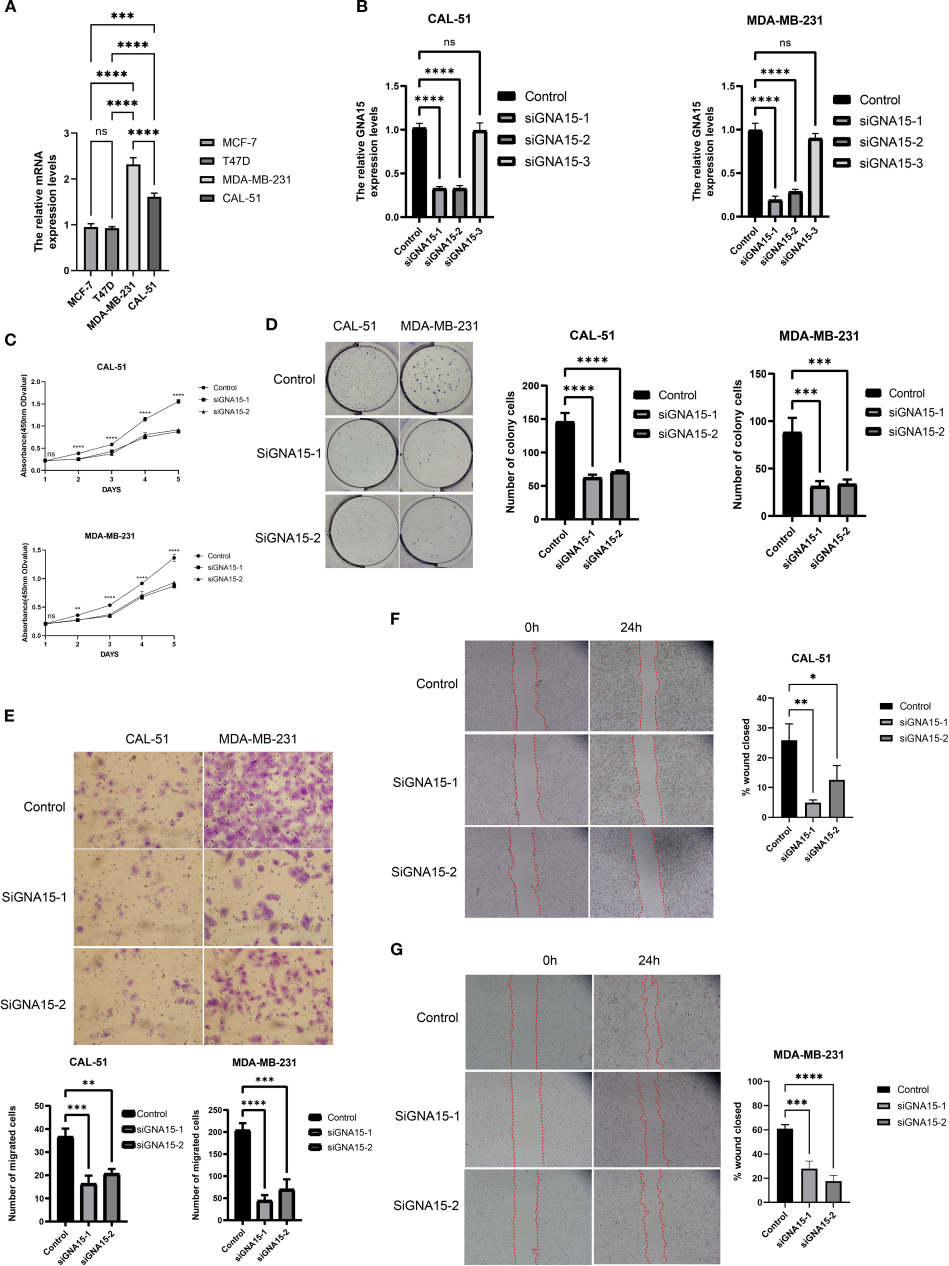
GNA15 was required for cell proliferation, migration, and invasion. **(A)** The mRNA level of *GNA15* was detected by RT–qPCR in breast cancer cell lines. **(B)** The efficiency of siRNAs was detected by RT–qPCR in CAL-51 and MDA-MB-231. **(C, D)** The cell proliferation assays were conducted, including CCK-8 **(C)** and colony formation assay **(D)**. **(E–G)** The invasion and migration assays were performed, including Transwell assay **(E)** and scratch assay **(F, G)**. ns, no significance; *p < 0.05; **p < 0.01; ***p < 0.001; ****p < 0.0001.

To further elucidate the biological role of *GNA15*, we conducted cell functional assays. Initial assessment by RT–qPCR confirmed elevated *GNA15* expression in TNBC cell lines (CAL-51 and MDA-MB-231), consistent with prior findings (**Figure 9A**). Using three specific siRNAs targeting *GNA15*, we identified two candidates that significantly reduced mRNA expression (**Figure 9B**). Subsequent CCK-8 and colony formation assays demonstrated that *GNA15* knockdown dramatically suppressed proliferative capacity and compromised malignant phenotypes in TNBC cells (**Figures 9C, D**). Furthermore, *GNA15* depletion substantially attenuated invasion rates in both cell lines (**Figure 9E**), while migration assays revealed significantly reduced migratory capacity in knockdown groups versus controls (**Figures 9F, G**). Considering the significant role of GNA15, we screened potential drugs for TNBC patients with high expression of GNA15 (**Supplementary Figure S5**). Taken together, these findings demonstrate that *GNA15* critically regulates TNBC proliferation, migration, and invasion.

## Discussion

Tumor heterogeneity is pervasive, driving differential therapeutic responses even within histologically uniform cancers (38). This heterogeneity stems from diverse origins—including cellular ontogeny, microenvironmental component infiltration, and aberrant intercellular communication (39). Triple-negative breast cancer, as the subtype of breast cancer with the poorest prognosis, displays more extensive tumor heterogeneity than hormone receptor-positive and HER2-positive breast cancer (7). Consequently, elucidating the mechanistic basis of TNBC heterogeneity is essential for personalized therapeutic strategies. Recent advances in single-cell sequencing and novel analytical frameworks have created unprecedented opportunities to resolve cellular heterogeneity and microenvironmental signaling dynamics (21, 22, 40). In this study, we employed multi-scale data, including single-cell and bulk RNA sequencing, to comprehensively delineate the extensive heterogeneity among tumor clusters. To the best of our knowledge, this constitutes the first identification of a tumor progression-associated cluster characterized by *GNA15* overexpression in TNBC.

Research on TNBC heterogeneity dates back over a decade. Recent classifications delineate four principal TNBC subtypes: immunomodulatory (IM), luminal androgen receptor (LAR), mesenchymal-like (MES), and basal-like immune-suppressive (BLIS) (9). Prognostically, IM subtypes demonstrate favorable outcomes, while MES subtypes exhibit poor survival—consistent with our findings where the S3 cluster aligned with IM characteristics and showed improved prognosis. The significant signaling pathways, including interferon-gamma (41), TNF-alpha (42), and *IL6-JAK-STAT3* (43), were reported to correlate with immunomodulatory approaches in the tumor microenvironment and were highly enriched in the S3 cluster. Concurrently, we identified the aggressive S2 cluster as a trajectory endpoint exhibiting mesenchymal characteristics: epithelial–mesenchymal transition, angiogenesis, and MYC target activation. Complementary GO and KEGG analyses confirmed the enrichment of the top 50 markers of the S2 cluster in cell growth, ECM–receptor interactions, and focal adhesion, collectively indicating a mesenchymal-like phenotype. While our single-cell clusters partially recapitulate established transcriptomic subtypes, we further observed divergent cell-cycle regulatory programs: one driven by MYC/p53 signaling and another by the E2F/G2M pathways. Notably, androgen-sensitive and basal subtypes were not distinctly segregated at single-cell resolution, suggesting fundamental differences between tissue-level heterogeneity and cellular heterogeneity. All in all, our study provides a new theoretical basis for revealing the heterogeneity of TNBC.

Given the adverse prognostic effect of S2, we decided to further investigate the underlying reasons driving its malignant features. The lineage plasticity of tumors has been proven to prompt tumor cells to adapt to the external environment and change accordingly, thereby enhancing their survival ability (34). In consideration of this, we mapped the potential lineage trajectories of the cells, and the results confirmed that the S2 cluster may have evolved through the lineage of tumor subgroups. However, multiple determinants contribute to perturbations in tumor cell biological activities and infiltrative heterogeneity. We sought to further elucidate the origin of malignant features and the invasive biological behavior of the S2 cluster from the perspective of the tumor microenvironment. Cell–cell interaction analyses demonstrated that the S2 cluster, acting as a receptor, displayed the strongest reception of ligand signals emanating from CAFs. Receptors involved in the FN1 and collagen pathways were significantly overexpressed in the S2 cluster. Receptors, including *CD44* (44), *SDC4* (37), and *ITGA2* (45), have been confirmed to be upstream molecules of malignant pathways. These findings revealed that the S2 cluster may be modulated by CAFs to further exhibit aggressive biological behaviors. Targeting the interaction molecules between CAFs and the S2 cluster may be a potential approach for the treatment of TNBC.

We further interrogated the co-expression gene network of the S2 cluster and identified *GNA15* as a critical hub gene. Herein, we report, for the first time, the role of *GNA15* in driving tumor progression in breast cancer, with *in vitro* experiments validating the findings of our bioinformatics analyses. While the molecular mechanism underlying *GNA15* action awaits further experimental validation, published studies lend support to our results. Zanini et al. (46) found that downregulation of *GNA15* inhibited cell proliferation in the small intestinal neuroendocrine neoplasia cell line and was associated with ERK, NF-kappaB, and Akt pathway signaling. Additionally, *GNA15* has been reported to facilitate the malignant development of thyroid carcinoma and acute myeloid leukemia via the MAPK signaling pathway (47, 48). Moreover, Innamorati et al. (49) found that *GNA15* could serve as a biomarker for early-stage pancreatic ductal adenocarcinoma. Several studies have also implicated *GNA15* in promoting tumor drug resistance. Luo et al. (50) confirmed that *GNA15* induces drug resistance in B-cell acute lymphoblastic leukemia via AMPK signaling. Li et al. (51) observed that *GNA15* was upregulated in cisplatin-resistant ovarian cancer cells. Our results revealed that knockdown of *GNA15* expression impairs the proliferation, invasion, and migration of triple-negative breast cancer cell lines. Bioinformatics analyses further indicated that *GNA15* is associated with the MAPK signaling pathway. Collectively, these findings identify *GNA15* as a hub gene for the S2 cluster and a potential therapeutic target for TNBC. In summary, single-cell and transcriptome data identified that the S2 subgroup affects the poor prognosis of triple-negative breast cancer, suggesting that the S2 subgroup may serve as a future therapeutic target for triple-negative breast cancer. Its infiltration abundance may serve as a potential indicator for monitoring the disease progression of triple-negative breast cancer. As the core gene of the S2 subgroup, GNA15, targeting this molecule or combining it with other breast cancer treatments in the future will provide more possible therapeutic strategies for triple-negative breast cancer.

Although this study comprehensively analyzed the heterogeneity of TNBC tumor cells by combining multiomics analysis and *in vitro* experiments, there are still some limitations. First, the limited number of bulk RNA samples may lead to analytical bias. More publicly available data would be incorporated to formally analyze the universality of the results. Second, the deconvolution algorithm was used to infer the prognosis of tumor subpopulations, which may not fully and accurately reflect the cell infiltration level of tumor subpopulations. Also, the *p*-values of survival analyses (the S2 and S3 clusters) are relatively borderline. Further multicenter studies are needed to confirm the prognostic role of the S2 cluster and *GNA15*. Third, the molecular mechanism of *GNA15* has not yet been confirmed. We plan to conduct *in vivo* and *in vitro* experiments (Western blotting, Co-Immunoprecipitation (CO-IP), mass spectrometry, etc.) in the subsequent research to further explain the regulatory relationship between *GNA15* and the MAPK signaling pathway.

## Conclusions

Our study comprehensively elucidates the extensive heterogeneity among TNBC tumor cells, encompassing transcription factors, meta-programs, cell lineage evolution, cell communication, co-expression networks, and prognostic profiles. Most notably, we identified that *GNA15*, the core gene of the poor prognosis-associated S2 subgroup, drives the progression of TNBC. These findings deepen our understanding of TNBC heterogeneity and lay a theoretical foundation for the development of therapeutic strategies for TNBC.

## Data Availability

The datasets presented in this study can be found in online repositories. The names of the repository/repositories and accession number(s) can be found in the article/**Supplementary Material**.
