# Supplementary material for: Multiomics profiling identifies the poor prognostic role of a tumor cluster with GNA15 overexpression in triple-negative breast cancer
Source: Front Immunol. 2025 Sep 22;16:1659183. doi: 10.3389/fimmu.2025.1659183 (PMC12497757; doi:10.3389/fimmu.2025.1659183)
Supplement: Supplementary file 2 [file Table2.docx]

**Supplementary Figure**

**
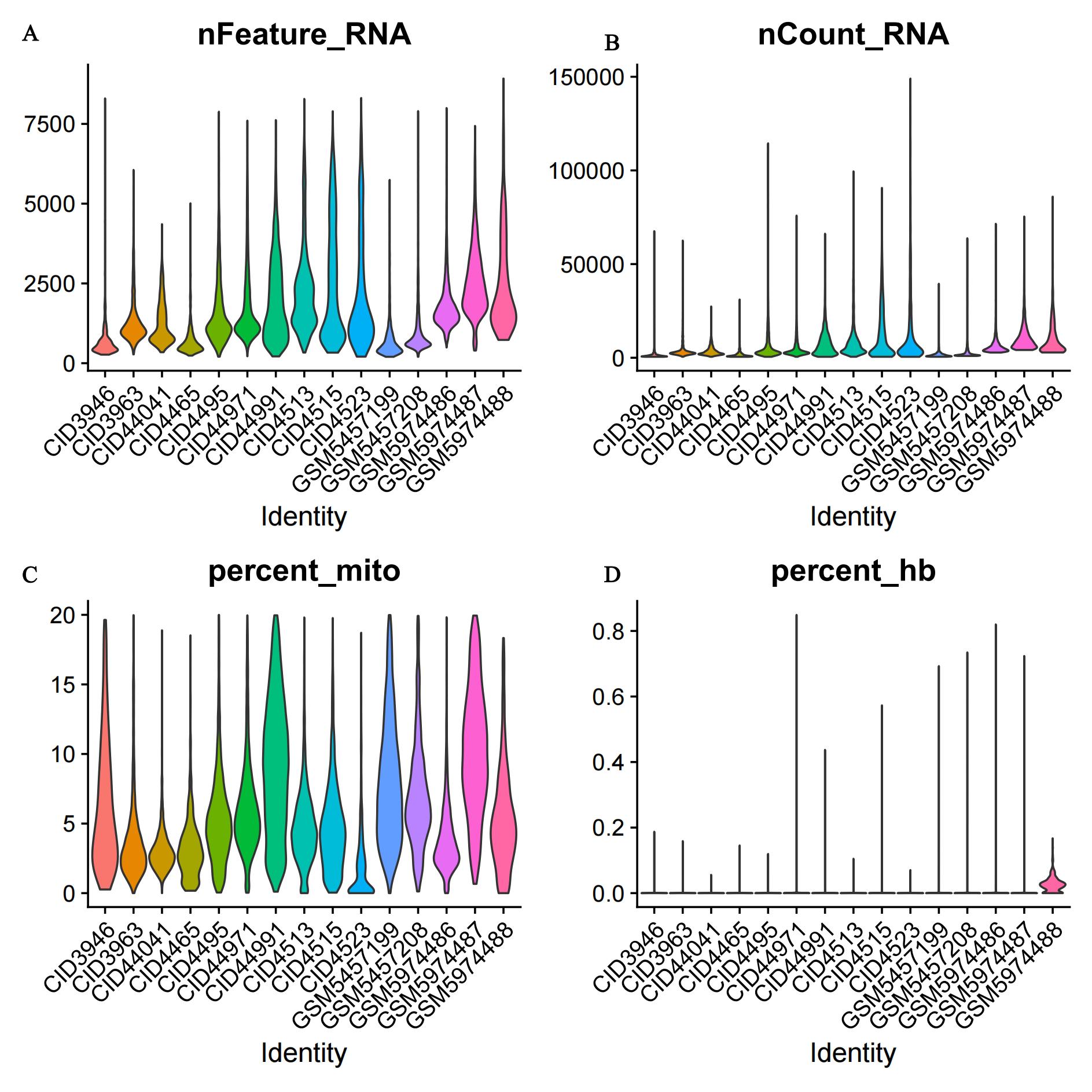
**

**Figure S1 The quality control of single-cell RNA sequencing (scRNA-seq) of TNBC samples. A-D** The violin plots showing the strict standard criteria of scRNA-seq in the number of genes detected per cell (**A**), total number of molecules detected per cell (**B**), proportion of mitochondrial genes(**C**), and proportion of hemoglobin genes (**D**).


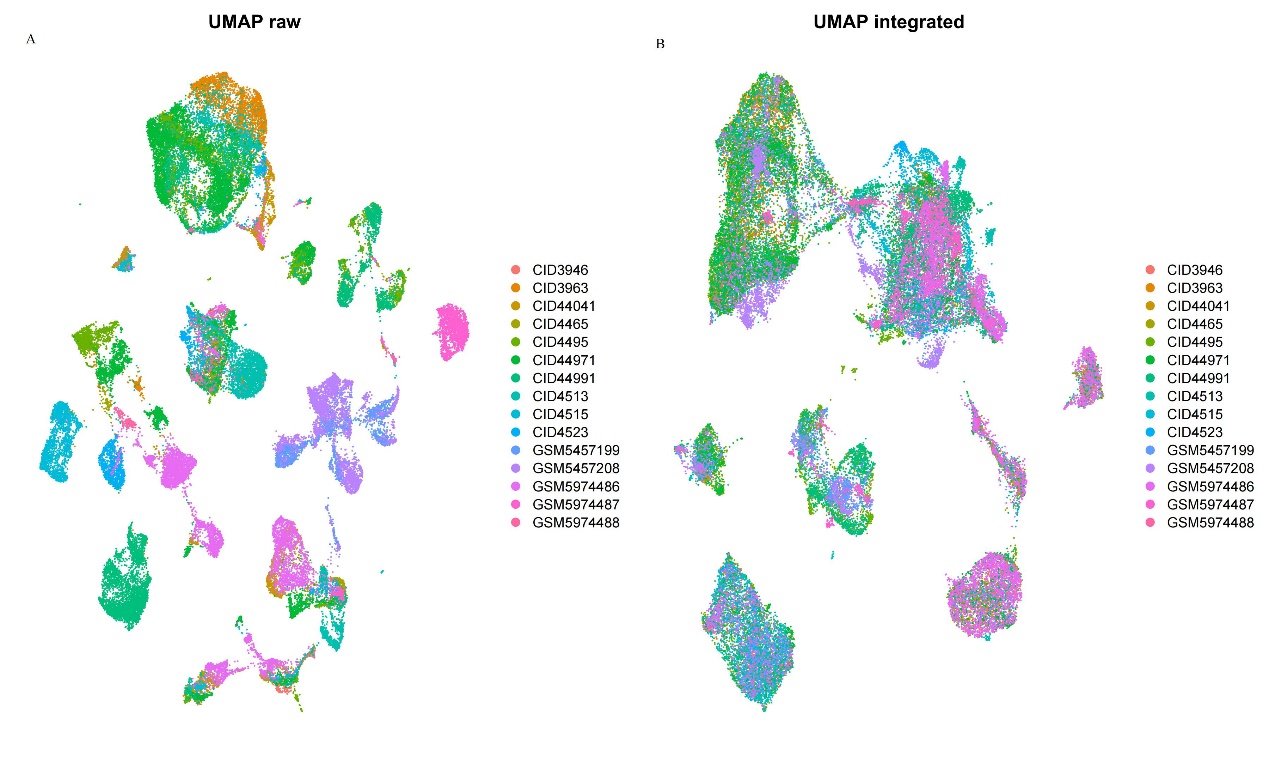


**Figure S2 Batch removal among TNBC samples.** UMAP scatter plots showed the batch effect was removed after using harmony algorithm.


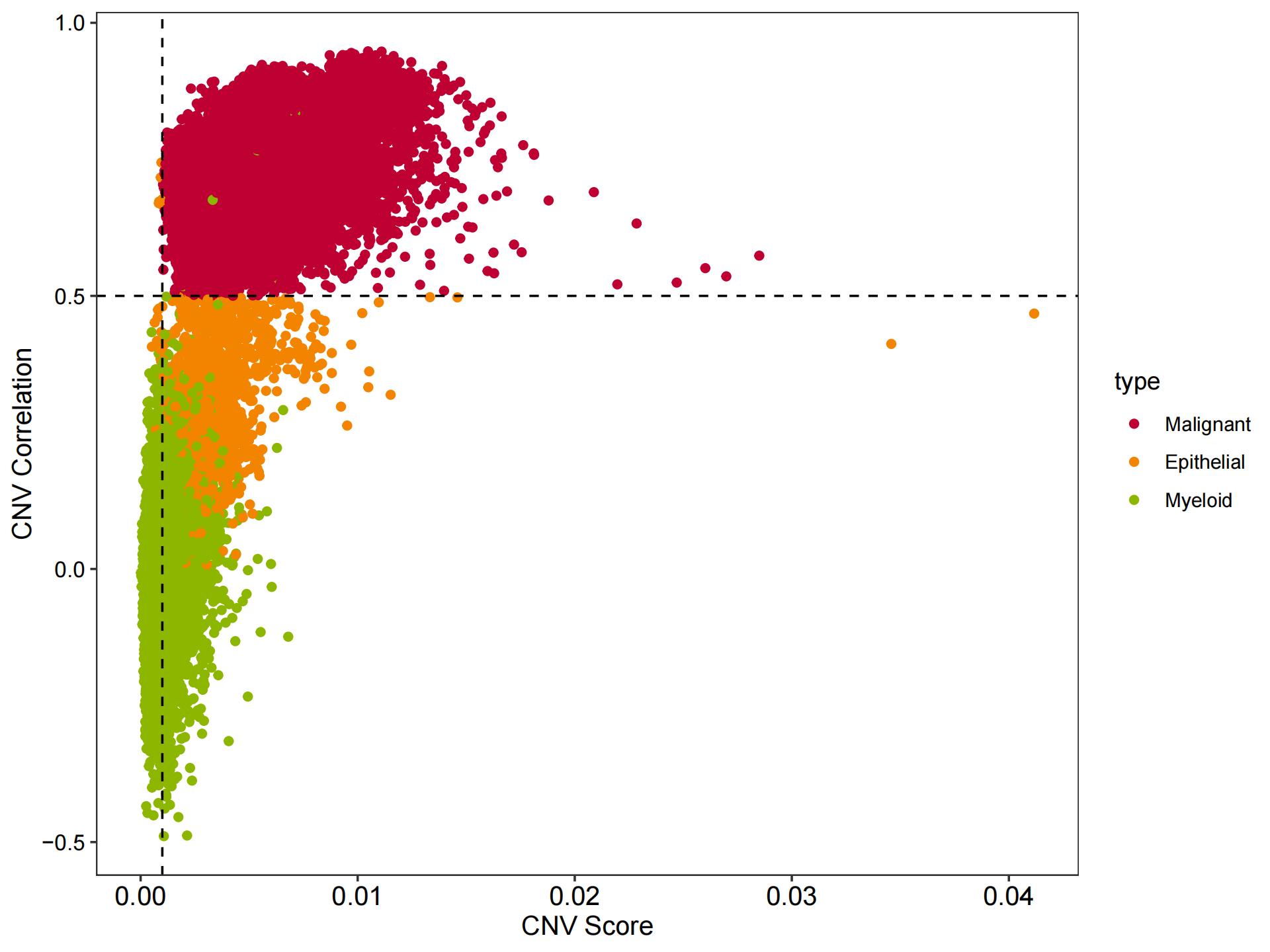


**Figure S3 The copy number variation of TNBC samples.** Scatter Plot showing the CNV correlation and CNV score of referenced cells and observed cells.


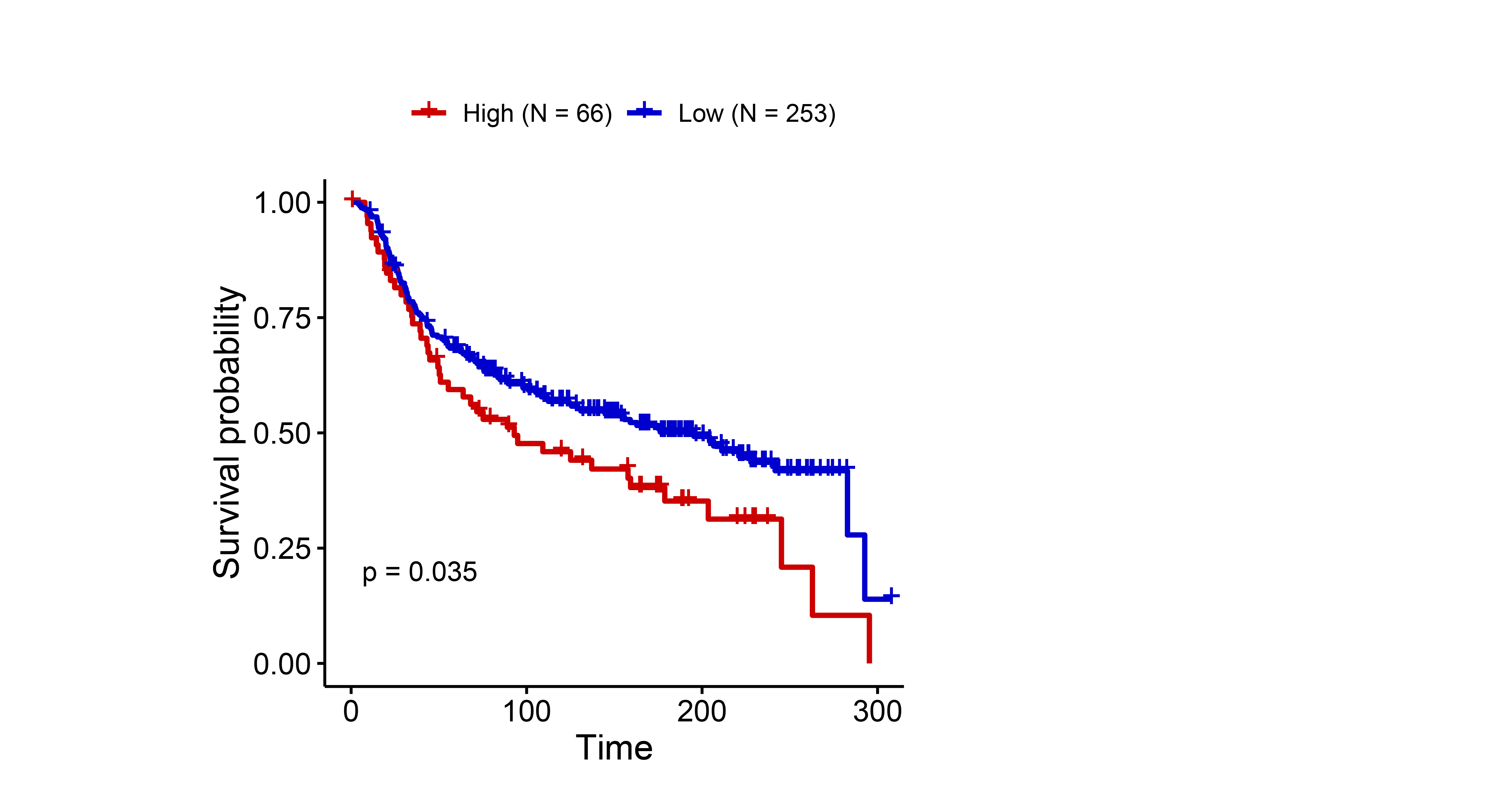


**Figure S4 The prognostic role of S2 cluster in TNBC.** The Kaplan-Meier curve of S2 cluster in METABRIC dataset.


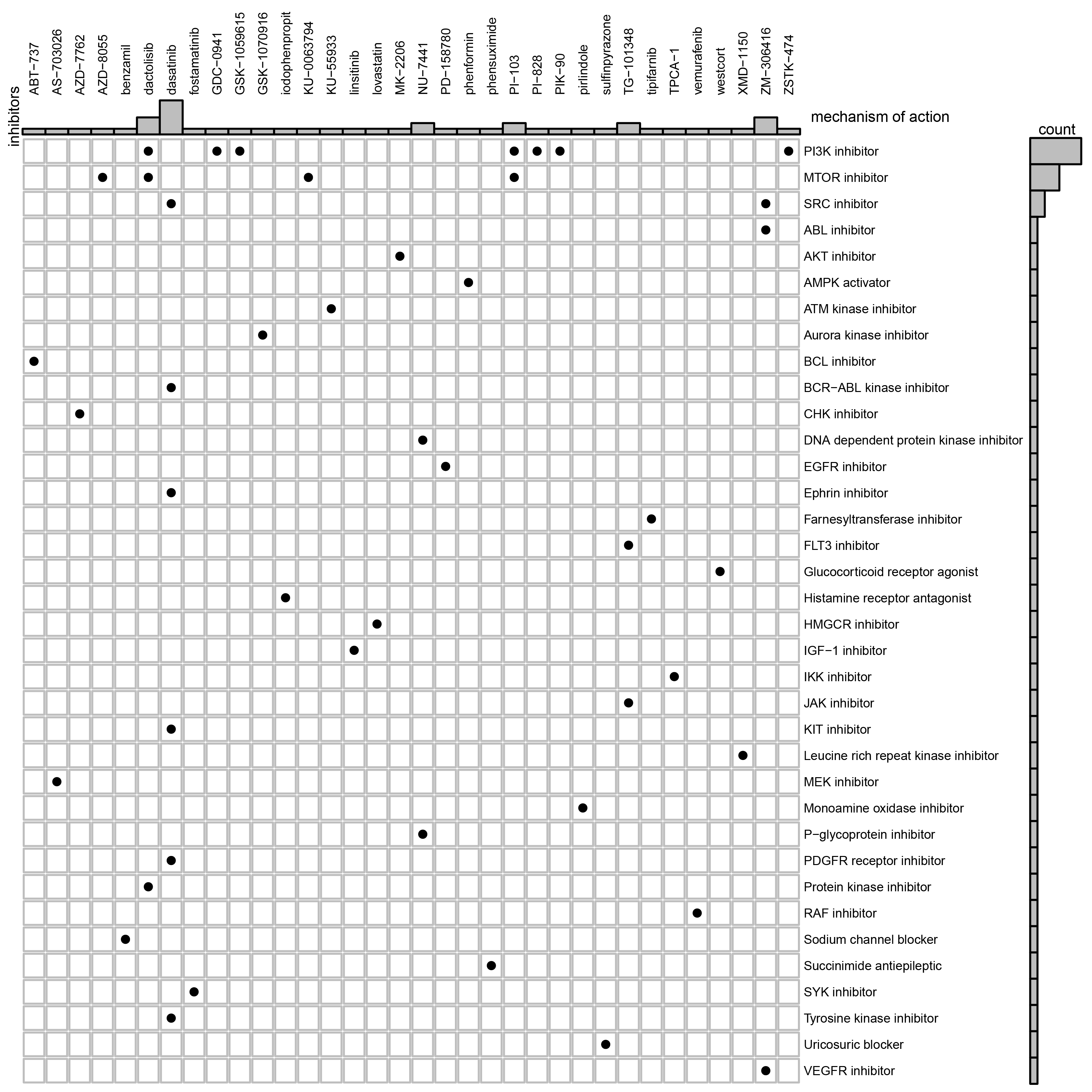


**Figure S5 Candidate inhibitors for S2 cluster.** The names and action of mechanism of potential drugs for high expression of GNA15 patients.
